# Low inter-examiner agreement of the Friedman staging system indicating limited value in patient selection

**DOI:** 10.1007/s00405-018-4970-z

**Published:** 2018-04-16

**Authors:** Joar Sundman, Johan Fehrm, Danielle Friberg

**Affiliations:** 1 Department of Clinical Science, Intervention and Technology, Karolinska institutet, 171 77 Stockholm, Sweden; 2 Department of Otorhinolaryngology, Karolinska University Hospital, 141 86 Stockholm, Sweden; 3 Institute of Surgical Science, Uppsala University, 751 85 Uppsala, Sweden

**Keywords:** Inter-examiner agreement, Friedman tongue position, Uvulopalatopharyngoplasty, Obstructive sleep apnoea

## Abstract

**Purpose:**

The Friedman staging system is a clinical method for selecting patients with obstructive sleep apnoea who are likely to benefit from uvulopalatopharyngoplasty. The objective of this study was to evaluate the system by determining its inter-examiner agreement.

**Methods:**

Twelve patients with obstructive sleep apnoea were examined by 14 doctors. The Friedman stage was derived from tonsil size and tongue position, and a Cohen’s kappa analysis was performed to assess inter-examiner agreement.

**Results:**

One hundred and sixty-eight ratings were performed. The median kappa for tongue position was 0.32 (first and third quartiles: 0.21 and 0.44) and was 0.62 (0.50 and 0.63) for tonsil size. The median kappa for the Friedman stage was 0.38 (0.24 and 0.55), which corresponds to only a slight or fair agreement.

**Conclusion:**

The Friedman staging system demonstrated a low inter-examiner agreement, indicating that the system is an uncertain method for selecting patients for uvulopalatopharyngoplasty.

**Level of evidence:**

2B.

## Introduction

The Friedman staging system is a clinical method for selecting patients with obstructive sleep apnoea (OSA) who are likely to benefit from surgical intervention with uvulopalatopharyngoplasty (UPPP). Friedman et al. demonstrated in 2002 that the system could predict outcomes after UPPP by scoring 134 patients with OSA into three different stages. Patients scored as stage I had an 81% success rate (defined as a respiratory distress index below 20 and reduced by at least 50%), stage II had a success rate of 38%, and stage III had a success rate of 8% [1]. Li et al. subsequently confirmed a similar correlation in 110 patients in 2006 [2]. At our department, the Friedman staging system is important in the decision of whether to recommend surgery or not. We also used it as an inclusion criterion in our randomized-controlled study SKUP^3^ from 2013, evaluating data from polysomnography after modified UPPP in 65 patients [3]. However, a recent study of success factors from all operated patients in SKUP^3^ showed that tonsil size and not Friedman stage was a predictor of success after 6 months [4], indicating that the method might be less accurate than previously expected.

As with any clinical method, the results of the Friedman staging system can be expected to vary among different examiners, thus affecting its inter-examiner agreement. Too much variation, however, limits the value of the method and questions its reliability.

**Fig. 1:**
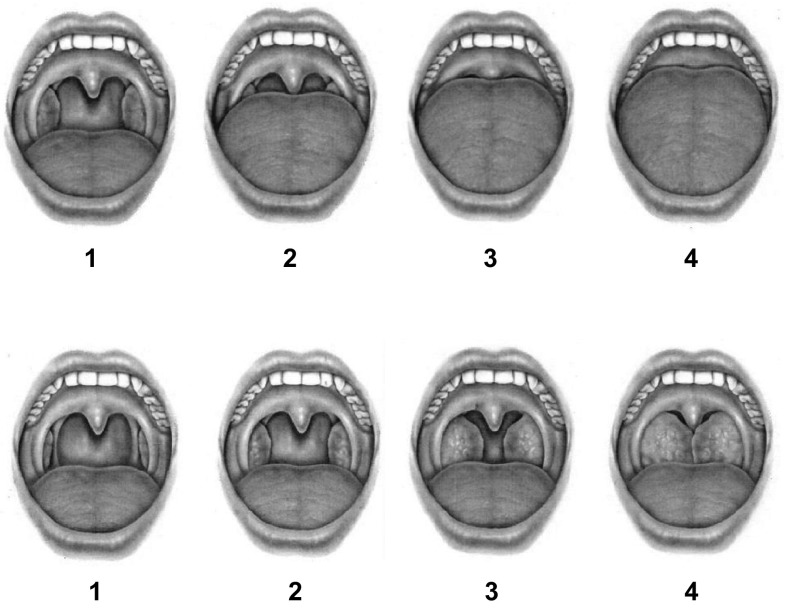
The four different Friedman tongue positions (first row) and tonsil sizes (second row). Courtesy of Professor Friedman and co-authors

The system was originally based on tonsil size and four different tongue positions (Fig. 1). Because the tongue and soft palate are mobile structures, it seems likely that, especially, the Friedman tongue position could vary between examinations. However, two previous studies found a high inter-examiner agreement with a kappa value of 0.83 and 0.93 using video clips of oropharyngeal examination [5, 6].

We hypothesized that this agreement would be lower when real-life subjects were examined, and we conducted a study in 2016 in which 15 doctors evaluated each other’s Friedman tongue position [7]. In this study, we found a median Cohen’s kappa coefficient of 0.36, which corresponded to only a slight [8] or fair [9] agreement.

This former study did have some major limitations though, mainly the fact that the raters were non-experts in using the Friedman staging system and the subjects did not have an OSA diagnosis. We, therefore, wanted to repeat the study in a more realistic setting, letting otorhinolaryngology (ORL) doctors examine patients with OSA who were possible surgical candidates and having them evaluate both the tonsil size and the Friedman tongue position to determine the more clinically relevant Friedman stage.

## Materials and methods

Twelve patients participated and were examined by 14 doctors, of whom two were experienced specialists in sleep medicine, nine were ORL specialists, and three were ORL residents. All had used Friedman staging system before, but to an extent varying from very often to more seldom. The patients gave their written consent to participate, and ethics approval was obtained from the Regional Ethical Review Board in Stockholm, 2015/755–31/2, supplement 2016/33–32. The study was conducted at the ORL department at Karolinska University Hospital in 2016. The conventional consulting rooms, headlights, and examination chairs were used. The doctor and patient were alone in the consulting room. We used the Friedman tongue position with four grades, previously known as the Friedman palate position or the modified Mallampati position. The instructions were given as in Friedman et al. [1], and the doctors asked the patient to open their mouth widely without protruding the tongue, repeated the procedure five times, and assigned what they believed to be the most accurate level of tongue position [1]. When evaluating tonsil size, a tongue depressor was allowed if necessary. The doctor then noted the findings on pre-printed templates, folded the paper, and kept it hidden from the patient and other doctors. The Friedman stage was calculated from tonsil size and Friedman tongue position, as explained in Table 1. All patients had a BMI < 40, thus not affecting the staging.

**Table 1:** Friedman stage from tonsil size and the Friedman tongue position

Stage	Friedman tongue position	Tonsil size
I	1, 2	3, 4
II	1, 2	1, 2
	3, 4	3, 4
III	3, 4	1, 2

The data were analysed with Cohen’s kappa, which measures the amount of inter-examiner agreement that occurs beyond what would be explained by chance alone. The range of kappa is usually between 0 and 1, where 0 represents agreement that would be expected from random chance, and 1 represents perfect agreement. Although unlikely, negative values (down to − 1) are possible and represent agreement that is even less than would be expected from random chance alone.

The software R was used for statistical computing [10] and a kappa coefficient was calculated for each possible pair of raters. The first and third quartiles, as well as the range, are presented, and standard interpretations of kappa according to Byrt [8] (poor < 0.20, slight 0.21–0.40, fair 0.41–0.60, good 0.61–0.80, very good 0.81–0.92, and excellent 0.93–1.00) and Altman [9] (poor < 0.20, fair 0.21–0.40, moderate 0.41–0.60, good 0.61–0.80, and very good 0.81–1.00) were used. The number of raters was chosen to be similar to the number in our previous study [7] (*n* = 15) and no power analysis was performed.

The Friedman tongue position was slightly modified in 2008 with added grades (2a and 2b) [5]. Because these extra grades have not been correlated to surgical outcome and because our department was familiar with the previous system, we used the Friedman tongue position with four grades.

## Results

Each of the 14 doctors rated tonsil size and Friedman tongue position in 12 patients, corresponding to a total of 168 ratings, with 14 individual series of ratings and 91 comparable pairs of raters. All the raters examined all the patients, and there were no missing values and no dropouts.

**Fig. 2:**
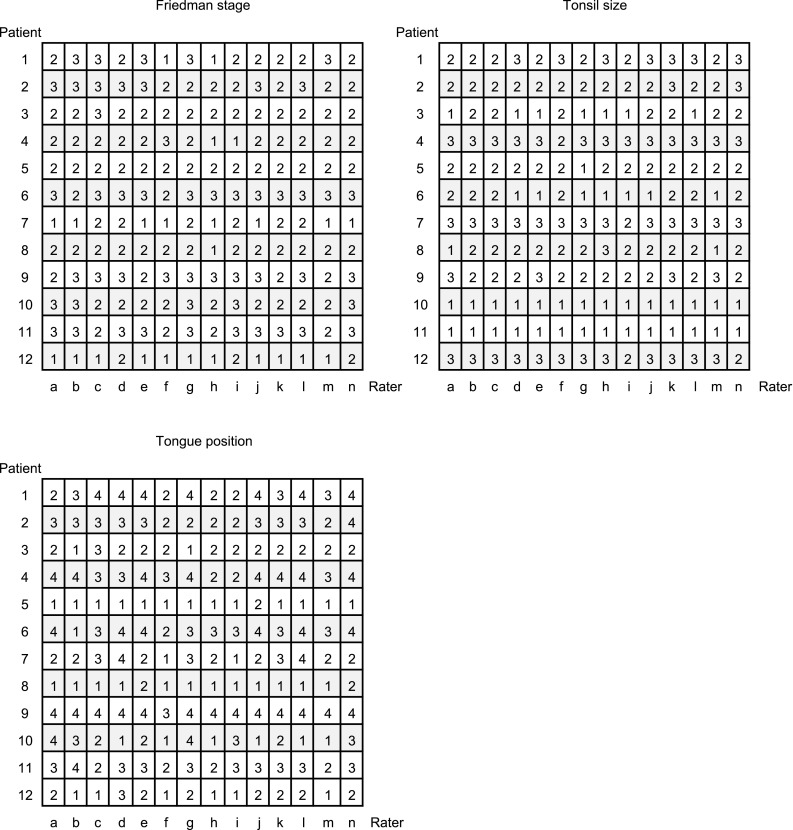
Results from all ratings; each patient 1–12, and each rater (doctor) a–n. Possible ratings for Friedman stage are 1, 2, or 3, and possible ratings for tongue position and tonsil size are 1, 2, 3, and 4. No patient was rated with tonsil size 4

The patients’ median age was 43 years (range 28–66 years), the median BMI was 26.9 kg/m^2^ (19.6–34.1 kg/m^2^), and 11 of the 12 patients were men. None had undergone any previous pharyngeal surgery. The results of all the ratings are shown in Fig. 2. The median Cohen’s kappa coefficient for tonsil size, tongue position, stage, and corresponding degree of agreement according to Byrt and Altman are given in Table 2.

**Table 2:** Results with median Cohen’s kappa values for the Friedman stage, tongue position, and tonsil size

	Cohen´s kappa (1st and 3rd quartile)	Range	Agreement (Byrt)	Agreement (Altman)
Friedman stage (*n* = 91)	0.38 (0.24, 0.55)	−0.08 to 0.86	Slight	Fair
Tongue position (*n* = 91)	0.32 (0.21, 0.44)	−0.09 to 0.77	Slight	Fair
Tonsil size (*n* = 91)	0.62 (0.50, 0.63)	0.14 to 1.00	Good	Good

Data are shown as the median with first and third quartiles, range, and the corresponding agreement according to Byrt and Altman. Cohen’s kappa is between − 1 and 1, where 0 represents agreement that would be expected from random chance, and 1 represents perfect agreement. Negative values represent agreement that is less than would be expected from random chance alone

*N* number of comparable pairs of ratings

## Discussion

In the present study, the doctors could not arrive at more than a slight or fair inter-examiner agreement of the tongue position (kappa 0.32) and staging (kappa 0.38) during live examinations of patients with OSA. This is in accordance with our previous study (kappa 0.36 for tongue position) and indicates that the method is uncertain.

The inter-examiner agreement on tonsil size was better, with a kappa of 0.62 (good agreement according to Byrt and Altman), and the poor outcome of the staging was, therefore, mainly due to difficulties in the agreement on tongue position. For example, in some patients, the doctors scored the same patient as having a tongue position of both the lowest and highest possible values. This high level of disagreement might be explained by differences in how the patients breathe during the examination. As suggested by Rodenstein et. al. [11], and easily observable during oral examination, the positions of the soft palate and tongue vary depending on whether the patient breathes through their nose or their mouth. While breathing through the nose, provided that the mouth is open, the soft palate will approach the tongue, thus favouring nasal airflow. In contrast, while breathing through the mouth, the soft palate will approach the posterior pharyngeal wall, favouring oral airflow. Consequently, rating of the Friedman tongue position with a patient breathing solely through their nose might give a higher value than a rating with the same patient breathing solely through their mouth. In the original description of how the Friedman staging is performed, as well as in the 2017 update [12], it is not specified whether the patient should breathe or not, nor whether to breathe through the nose or mouth. We believe that this needs to be specified, along with renewed investigation of the correlations with surgical outcomes. In our opinion, breathing through the mouth gives the most reproducible findings.

The strength of the present study is the realistic setting, with doctors examining adult patients with OSA of typical age, sex, and BMI using the conventional consulting rooms and equipment. Another strength, compared to the previous study using video clips, is that the repeated examinations captured the variations within the same patient at different times.

There are, however, several limitations to this study. First, although all participating doctors were familiar with the Friedman staging system, only two were strictly subspecialized in sleep medicine. This might have affected the results. However, all doctors received similar instructions with text and figures, and were asked to follow these strictly. A subgroup analysis of the participating doctors could possibly determine this, but was considered to be of limited value due to the differences in the number of comparable pairs (36 for specialists, 3 for residents, and only 1 for specialists in sleep medicine).

Second, a conventional Cohen’s kappa analysis does not differentiate in how inaccurate a rating is. This means that it is perceived as equally incorrect to score 1 and 2 as it is to score 1 and 4. This is sometimes statistically adjusted with a “weighted” kappa. However, the primary outcome, the Friedman stage, is ordinal data and consists of only three grades, and a weighted kappa was, therefore, not considered suitable. Third, there is no overall consensus of how Cohen’s kappa should be interpreted. A value considered low in one setting could perfectly well be acceptable in another. In our results, we attached two different interpretations. The interpretation by Altman was attached, since this is the most commonly used one. The interpretation by Byrt was added for comparison reasons, since it was used in the original study from 2008 of the inter-examiner agreement of Friedman tongue position.

One could argue that, because the study was conducted at a university hospital with doctors at different levels of training, the poor agreement might be a local problem and ungeneralizable and that the results might have been better at a clinic strictly focused on sleep surgery. Even though this might be true, it cannot be excluded that even experienced sleep surgeons might vary in their preoperative staging, consequently, giving different advice to their patients.

Finally, in favour of the Friedman staging system, it should be stressed that the alternatives are few, time-consuming, and still not evidence-based. For example, drug-induced sleep endoscopy has so far failed to show robust evidence for predicting pharyngo-surgical outcome [13, 14], although positive reports have shown its potential in the selection of patients before upper airway nerve stimulation [15]. Cephalometry was recently evaluated by Li et al., and, among 6 variables, only the distance from the hyoid bone to the mandibular plane showed a significant difference between patients who responded to UPPP and those who did not [2]. This demonstrates that patient selection for UPPP remains challenging and is a field in need of further research.

## Conclusion

The Friedman staging system had a slight or fair inter-examiner agreement among ORL doctors examining patients with OSA. We believe that further studies are needed, as well as clarity on whether the patient should breathe or not, and if so, whether through the nose or mouth. Meanwhile, the Friedman staging system should be used carefully in selecting patients for surgery, with respect to the staging system being more uncertain than previously known or published by others.
